# Supplementary material for: Small form factor implantable neural probe with efficient flip chip µLED for in vivo optogenetics
Source: Biomed Microdevices. 2025 May 29;27(2):24. doi: 10.1007/s10544-025-00754-1 (PMC12122578; doi:10.1007/s10544-025-00754-1)
Supplement: Supplementary file 1 — Supplementary Material 1 (DOCX 2.08 MB) [file 10544_2025_754_MOESM1_ESM.docx]

**SUPPLEMENTARY INFORMATION**


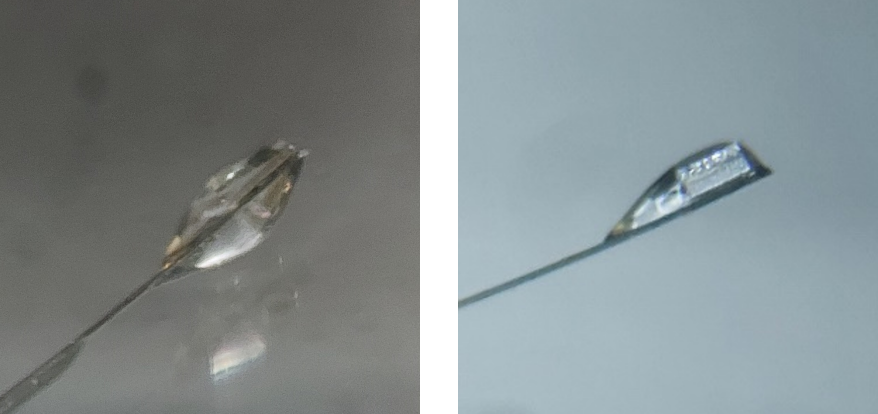


Sup. Figure 1. Comparison of coating applied to the probe with µLED between dip coating application (left) and our developed method on a pick-and-place machine (right). Dip coating application results in excessive coating volume on both sides of the neural probe and does not allow for control of final coating thickness. Total probe thickness after dip coating was estimated to be approximately 300 µm versus 100-105 µm with our novel method.

**
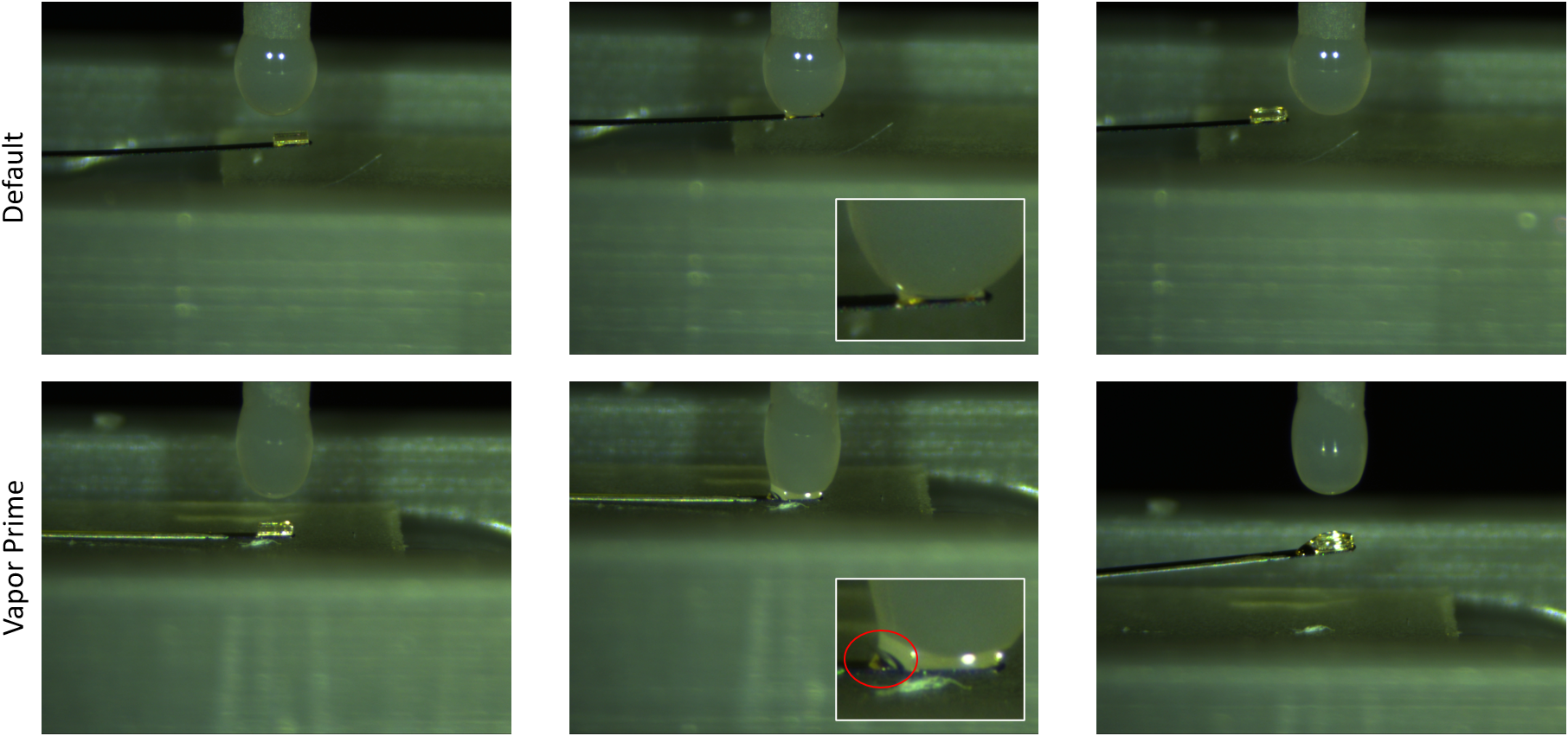
**

Sup. Figure 2. Exploratory preliminary test of hexamethyldisilazane (HMDS) vapor priming effect on passivation coating. Vapor priming increased the surface wettability and appeared to improve the coating application on top of the µLED and the underfilling between the µLED and the substrate (bottom panels), when compared with the standard method describe (top panels).
